# Recent Advances in Understanding Amino Acid Sensing Mechanisms that Regulate mTORC1

**DOI:** 10.3390/ijms17101636

**Published:** 2016-09-29

**Authors:** Liufeng Zheng, Wei Zhang, Yuanfei Zhou, Fengna Li, Hongkui Wei, Jian Peng

**Affiliations:** 1 Department of Animal Nutrition and Feed Science, College of Animal Science and Technology, Huazhong Agricultural University, Wuhan 430070, China; zhenglf2013@webmail.hzau.edu.cn (L.Z.); pjm@webmail.hzau.edu.cn (W.Z.); zhouyuanfei@mail.hzau.edu.cn (Y.Z.); 2 The Cooperative Innovation Center for Sustainable Pig Production, Wuhan 430070, China; 3 Scientific Observing and Experimental Station of Animal Nutrition and Feed Science in South-Central, Ministry of Agriculture, Hunan Provincial Engineering Research Center of Healthy Livestock, Key Laboratory of Agro-ecological Processes in Subtropical Region, Institute of Subtropical Agriculture, Chinese Academy of Sciences, Changsha 410125, China; lifengna@isa.ac.cn

**Keywords:** mTORC1, amino acids, membrane transceptor, membrane receptor, sensor

## Abstract

The mammalian target of rapamycin (mTOR) is the central regulator of mammalian cell growth, and is essential for the formation of two structurally and functionally distinct complexes: mTORC1 and mTORC2. mTORC1 can sense multiple cues such as nutrients, energy status, growth factors and hormones to control cell growth and proliferation, angiogenesis, autophagy, and metabolism. As one of the key environmental stimuli, amino acids (AAs), especially leucine, glutamine and arginine, play a crucial role in mTORC1 activation, but where and how AAs are sensed and signal to mTORC1 are not fully understood. Classically, AAs activate mTORC1 by Rag GTPases which recruit mTORC1 to lysosomes, where AA signaling initiates. Plasma membrane transceptor L amino acid transporter 1 (LAT1)-4F2hc has dual transporter-receptor function that can sense extracellular AA availability upstream of mTORC1. The lysosomal AA sensors (PAT1 and SLC38A9) and cytoplasmic AA sensors (LRS, Sestrin2 and CASTOR1) also participate in regulating mTORC1 activation. Importantly, AAs can be sensed by plasma membrane receptors, like G protein-coupled receptor (GPCR) T1R1/T1R3, and regulate mTORC1 without being transported into the cells. Furthermore, AA-dependent mTORC1 activation also initiates within Golgi, which is regulated by Golgi-localized AA transporter PAT4. This review provides an overview of the research progress of the AA sensing mechanisms that regulate mTORC1 activity.

## 1. Introduction

Mammalian target of rapamycin (mTOR) is an evolutionary conserved serine/threonine protein kinase, which shares significant homology with phosphatidylinositol kinase. It is essential for the formation of two functionally different complexes known as mTOR complex 1 (mTORC1) and mTORC2. mTORC1 is a conserved multi-protein complex which can regulate protein translation by controlling the phosphorylation of its downstream targets, the ribosomal protein S6 kinase (S6K) and eukaryotic translation initiation factor 4E binding protein (4E-BP1) [1]. Besides mTOR, mTORC1 contains four other components: regulatory-associated protein of mTOR (Raptor, a yeast Kog1 homolog) [2,3], mammalian lethal with Sec13 protein 8 (mLST8, also known as GβL) [4], dishevelled, Egl-10 and pleckstrin (DEP) domain containing mTOR interacting protein (Deptor) [5], and proline-rich Akt substrate 40 kDa (PRAS40) [6]. Raptor is an essential component for mTORC1 signaling, in which it functions as a scaffolding protein to recruit mTORC1 substrates [7]. Raptor also plays an important role in intracellular localization of mTORC1, leading to the activation of mTORC1 in response to amino acid stimulation [8]. mLST8 is a common component of both mTORC1 and mTORC2, and may contribute to their stability and activity. PRAS40 was originally identified as a substrate of Akt kinase [9], but following studies showed that it may also be a negative regulator of mTORC1 [6]. Deptor is an inhibitor of mTOR, which directly binds to mTOR and negatively regulates the functions of both mTORC1 and mTORC2 [5].

**Figure 1:**
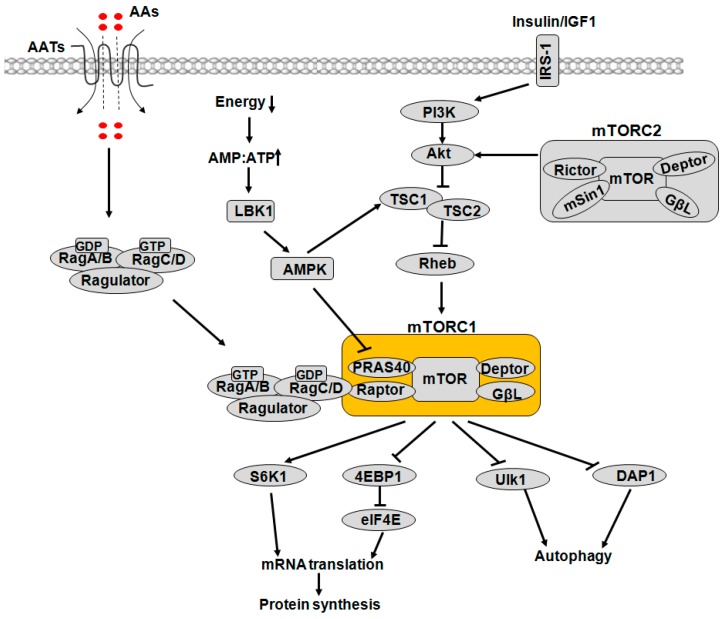
Model of mTOR signaling network in mammalian cells. It consists of two functionally different complexes known as mTORC1 and mTORC2. The mTORC1 pathway integrates inputs from at least three major cues, namely AAs, growth factors (such as IGF1 and insulin) and energy status, to regulate many major processes, including protein synthesis and autophagy. mTORC1 signaling is highly sensitive to AAs, which can be transported into cells through plasma membrane AATs, and then exert their regulatory roles. AA-dependent activation of mTORC1 requires small Rag GTPases. There are four Rag proteins that work in heterodimers. Rag A or B binds to Rag C or D. Upon growth factor stimulation, mTORC1 signaling is activated through the classical PI3K-PKB/Akt-TSC-Rheb pathway. Energy status is also sensed upstream of mTORC1. Low energy activates AMPK, which inhibits mTORC1 function by phosphorylating and activating TSC2 as well as phosphorylating Raptor. Arrows represent activation, whereas bars represent inhibition. mTOR: mammalian target of rapamycin; mTORC1: mTOR complex 1; IGF1: insulin-like growth factor 1; AAs: amino acids; AATs: AA transporters; PI3K: phosphatidylinositide 3-kinase; PKB/Akt: protein kinase B; TSC: tuberous sclerosis complex; Rheb: Ras homolog enriched in brain; Raptor: regulatory-associated protein of mTOR; AMPK: AMP-activated protein kinase.

mTORC1 is a central cell growth controller that integrates the signaling of growth factors, nutrients and energy supplies to control biosynthetic and catabolic processes [1]. Regulation of mTORC1 by most signals occurs primarily through two types of mechanisms: (1) direct modification of mTORC1 components; and (2) regulation of the upstream factors, including the Ras-related guanosine triphosphatases (Rag GTPases) and Ras-homolog enriched in brain (Rheb) [8,10,11] (Figure 1).

The tuberous sclerosis complex 1/2 (TSC1/2) appears to be a key signal integration factor, which accepts growth factor signaling and functions as a GTPase-activating protein (GAP) towards Rheb, a compulsory activator of mTORC1. The growth factor signaling involves phosphatidylinositide 3-kinase (PI3K)-protein kinase B (PKB, also known as Akt), p90 ribosomal S6 kinase 1 (RSK) and extracellular-signal-regulated kinase 1/2 (ERK1/2) [12]. Besides, PKB/Akt can also phosphorylate PRAS40 to dissociate it from mTORC1 [13]. The cellular energy status or glucose availability affects mTORC1 through AMP-activated protein kinase (AMPK), which acts as the crucial energy sensor and is activated by a fall in ATP (concomitant with a rise in ADP and AMP) [14]. Under nutrient starvation, AMPK serves as a negative regulator of mTORC1 through direct phosphorylation of TSC2 and Raptor, thus inhibiting cell growth and preserving energy [15].

Amino acids (AAs) not only act as substrates for protein synthesis and intermediates in lipid and adenosine triphosphate synthesis, but also directly initiate a signaling cascade leading to the activation of mTORC1. AA deprivation causes a dramatic decline in the phosphorylation of S6K and 4E-BP1, which cannot be compensated by other stimuli such as growth factors or energy [11,16], indicating that AAs are required for mTORC1 activation. Furthermore, the mechanisms by which growth factors and energy stimulate mTORC1 activity have been extensively studied. However, how AAs are sensed and signal to mTORC1 is not fully understood. One critical question is from where AA signaling that regulates the activity of mTORC1 initiates. The objective of this article is to review recent advances in the studies about the AA sensing mechanisms that regulate mTORC1.

## 2. Regulation of mTORC1 by Amino Acids

Barbet et al. [17] reported that starved *Saccharomyces cerevisiae* showed a profound inhibition of overall mRNA translation, and behaved like the cells treated with rapamycin or depletion of both TOR1 and TOR2. After that, researchers found that nutrients could also mediate TOR signaling, and revealed that it is AAs that could regulate mTOR signaling independently of insulin [16]. Depletion of AAs from the culture medium profoundly inhibited S6K activation and 4E-BP1 phosphorylation, which became insensitive to all agonist treatments. Phosphorylation and responsiveness of S6K and 4E-BP1 to insulin were quickly restored following the reintroduction of AA mixture [16]. Among AAs, leucine, glutamine and arginine are the key mediators of mTORC1 [18,19,20,21]. The deprivation of leucine suppresses mTORC1 activation, and thus rapidly inhibits the phosphorylation of both S6K and 4E-BP1 [3]. The stimulation with almost all essential AAs, particularly leucine, would increase the phosphorylation of ribosomal s6 kinase (p70S6K1) [22]. Besides, glutamine could coordinate with leucine to regulate the activation of mTORC1 by inhibiting the activity of AMPK [23] and promoting glutaminolysis and a-ketoglutarate production [21]. Furthermore, Yao et al. [24] reported that the addition of arginine to diet could increase the activity of mTORC1 and synthesis of skeletal muscle protein in neonatal pigs.

It is still unclear how mammalian cells perceive the prevailing amino acid availability and relay this information to signaling pathways within the cell. There are two main possibilities. Firstly, mammalian cells may possess a plasma-membrane AA sensor similar in concept to the yeast sensor Ssy1p [25], which detects extracellular AA levels. Alternatively, mammalian cells may possess an intracellular AA sensor, which can initiate specific signaling to mTORC1.

## 3. Amino Acid Signaling to mTORC1 Initiates inside the Cells

Beugnet et al. [26] identified that it was intracellular AA availability that could regulate the activity of mTORC1. AAs should be transported into cells, and then exert their regulatory roles. Hence, it seems that AA signaling that regulates the activity of mTORC1 should initiate inside the cells. As the gate-keepers, AA transporters are usually responsible for the exchange of AAs between intracellular and extracellular medium. These facts indicate that AA transporters responsible for the transport of leucine, arginine and glutamine may play an important role in the regulation of mTORC1. The transport of leucine is usually performed by system L AA transporter 1 (LAT1) [27], while the influx of glutamine is mostly accomplished by system ASC AA transporter 2 (ASCT2) [28] or sodium-coupled neutral AA transporter-2 (SNAT2) [29]. Although there is no evidence showing that the substrates of proton-assisted AA transporter 4 (PAT4) have the ability to affect the activity of mTORC1, previous researches have suggested that the expression of PAT4 is associated with mTORC1 activation [30]. Taylor et al. [31] clearly defined these AA transporters as “transceptors”, because they have dual transporter-receptor function (i.e., binding or translocation of AAs is coupled to the activation of an intracellular signaling cascade), which enables them to sense extracellular AA availability.

### 3.1. LAT1/SLC7A5

As we know, branched-chain AAs, especially leucine, are highly effective activators of mTORC1 among all AAs [18,19], and the system L AA transporter, including LAT 1/2 and the heavy chain of 4F2 cell surface antigen (4F2hc, also known as CD98), is a primary route for cellular entry of neutral AAs such as leucine [27]. LAT1-4F2hc and LAT2-4F2hc are obligatory exchangers to transport some large neutral AAs such as leucine into the cell to exchange with some intracellular AAs [32]. LAT1 is a highly expressed L-type AA transporter in a variety of cancer types, and the decrease of LAT1 expression inhibits cancer cell growth by inducing an intracellular depletion of neutral AAs such as leucine [33]. Inhibition of system L AA transporter leads to the decrease of leucine uptake and the phosphorylation of p70S6K [34].

### 3.2. ASCT2/SLC1A5

**Figure 2:**
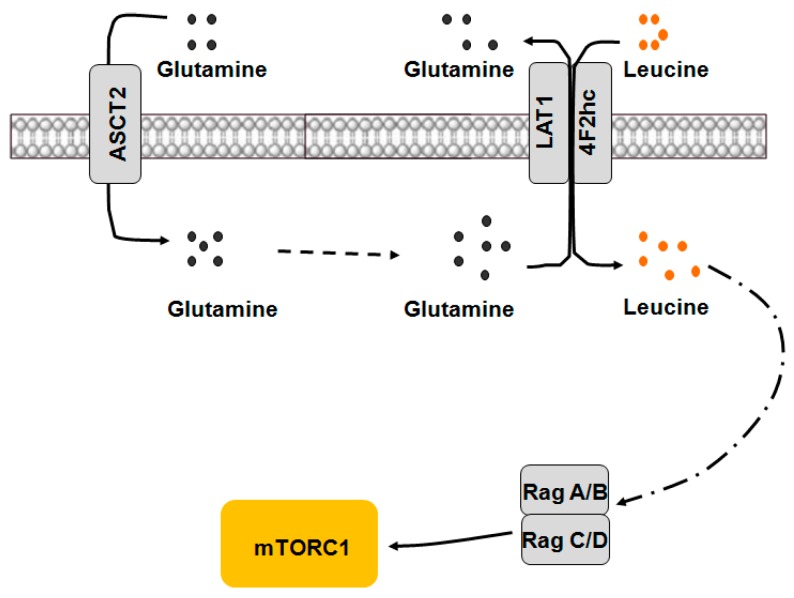
Bidirectional transport of AAs regulates mTORC1 activity. ASCT2 transports glutamine into the cell to increase its intracellular concentration. LAT1-4F2hc is a bidirectional transporter that mediates the simultaneous efflux of glutamine out of cells and uptake of extracellular leucine, which in turn activates mTORC1 signaling. AAs: amino acids; mTORC1: mammalian target of rapamycin complex 1; ASCT2: system ASC AA transporter 2; LAT1: system L amino acid transporter 1; 4F2hc: heavy chain of 4F2 cell surface antigen.

ASCT2, a Na^+^-dependent neutral AA transporter encoded by *SLC1A5*, effectively mediates glutamine efflux in exchange for small neutral AAs in the extracellular solution [35]. Fuchs and Bode [28] reported that ASCT2 is responsible for the influx of glutamine and maintains intracellular glutamine concentration, while LAT1 mediates the uptake of essential AAs by exchange with intracellular glutamine to meet the demand of metabolism and to regulate mTORC1. It has been shown that the cellular uptake of glutamine and its subsequent rapid efflux by obligatory exchange with essential AAs are required for mTORC1 activation [36]. Repression of ASCT2 decreases the activity of mTORC1 in cancer cells. Therefore, a bidirectional transport mechanism by which AAs regulate mTORC1 was proposed: firstly, ASCT2 transports glutamine into cells to increase the intracellular concentration of glutamine; and secondly, LAT1-4F2hc functions as a transceptor to mediate the simultaneous efflux of glutamine out of cells and uptake of extracellular leucine, subsequently leading to mTORC1 activation (Figure 2).

### 3.3. SNAT2/SLC38A2

SNAT2, which is encoded by the *SLC38A2* gene, belongs to the system A AA transporter family. SNAT2 is a Na^+^-dependent neutral AA transporter, and its substrates include glutamine, asparagine, alanine, serine, proline and glycine, etc. [29]. Knockdown of SNAT2 by ceramide caused a marked reduction in protein synthesis due to the reduced activation of the translation initiation factors downstream of mTORC1, which provides the first evidence that SNAT2 is important in AA-induced activation of mTORC1 [37]. AA deprivation increases SNAT2 expression [38], while insulin induces an increase of the plasma membrane abundance of SNAT2 in a PI3K-dependent manner [39]. Evans et al. [40] showed that SNAT2 inhibition in L6 muscle cells caused the depletion of cellular glutamine, which resulted in the depletion of other AAs, especially leucine, and subsequently reduced mTORC1 activation. Furthermore, SNAT2 was also proposed as a potential AA transceptor capable of sensing and signaling AA availability to mTORC1 [41,42]. However, the mechanisms by which SNAT2 monitors extracellular AA availability and regulates mTORC1 have not been clarified.

Although SNAT2 has overlapping substrate specificity with ASCT2, its function as the glutamine transporter is unique. ASCT2 mediates the uptake of glutamine, and then acts as an exchange substrate to accumulate leucine via LAT1-4F2hc under sufficient AA levels [43]. However, the SNAT2 mRNA level is low under normal physiological AA levels, whereas AA deprivation induces the translation of abundant SNAT2 mRNA by increasing the phosphorylation of eukaryotic initiation factor 2, leading to increased AA uptake [44]. This may explain the recent finding that mTORC1 activity is contrarily increased during glutamine starvation [45]. Additionally, SNAT1 and SNAT2 function as the complements to ASCT2 and proficiently provide exchange substrates to LAT1, thereby resulting in the normal mTORC1 activity in ASCT2 (−/−) cells [43].

### 3.4. PAT4/SLC36A4

As a member of proton-assisted AA transporters (PATs), PAT4 is responsible for a relatively high-affinity uptake of proline and tryptophan in an electroneutral Na^+^-independent manner. The pH dependence of PAT4 is distinct from other members of PATs: it has the maximal transport rate at pH 7.4 and a lower transport rate at pH 5.5 and pH 8.4 [46].

Of note, PAT4 is required for the activation of mTORC1, and thus is critical for mTORC1-regulated growth [30]. Moreover, it has been shown that small GTPase Rab12 promotes constitutive degradation of PAT4 and subsequently modulates mTORC1 activity and autophagy [47,48]. Rab12 is a member of the small GTPase Rab family that functions as a common regulator of membrane traffic in all eukaryotes [49]. Detailed colocalization analyses indicated that PAT4 colocalizes well with Rab12 at the recycling endosomes. Knockdown of Rab12 up-regulates PAT4 protein in the membrane. Thus, the authors suggested that Rab12 regulates the fusion of endosomes and lysosomes, whilst PAT4 in lysosomes is degraded. In addition, PAT4 over-expression leads to a result similar to that of Rab12 knockdown, whilst PAT4 knockdown offsets the increase in mTOR activity resulting from Rab12 knockdown [48].

Furthermore, the addition of AAs partially restored the p-S6K1 level in Rab12-overexpressing cells. Based on these results, Matsui and Fukuda [48] proposed a mechanism by which PAT4 participates in mTOR regulation. They suggested that PAT4 regulates intracellular AA levels (or may sense AAs); Rab12 controls the traffic of PAT4 between the membrane and lysosomes under normal conditions; and PAT4 is trafficked to lysosomes and is degraded, which results in the decrease of intracellular AA levels and inhibition of mTORC1. When Rab12 is knocked down, more PAT4 protein accumulates at the plasma membrane, augmenting AA uptake and enhancing mTORC1 activity. More recently, in HCT116 colorectal cancer cells, PAT4 was found to respond to two rapidly metabolized AAs (glutamine and serine) to drive mTORC1 activation by interacting with Rab1A (a small GTPase) and mTORC1 on the Golgi [50]. Since glutamine and serine bind to PAT4 with a lower affinity, and can compete with high-affinity PAT4 substrates [46], PAT4-mediated activation of mTORC1 may not depend on the transport function of PAT4.

## 4. Amino Acid Signaling to mTORC1 Initiates at the Lysosomes

**Figure 3:**
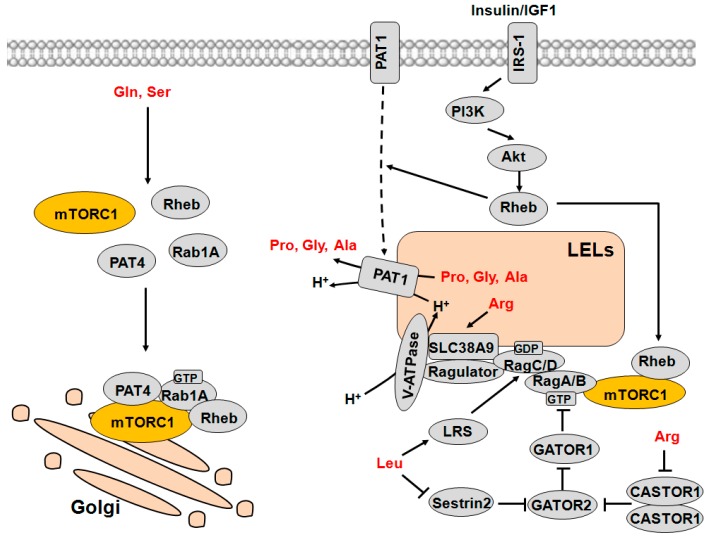
Model of AA-dependent activation of mTORC1 from LELs and Golgi apparatus. Multiple sensors are involved, including transporters (PAT1, PAT4 and SLC38A9) and cytosolic sensors (LRS, Sestrin2 and CASTOR1), which can sense specific AAs to activate mTORC1 on the surface of LELs or Golgi. Classically, mTORC1 is activated by AAs at LELs. Leucine activates Rag-mTORC1 by binding to LRS, which activates LRS GAP activity towards RagD, and by binding to Sestrin2, which inhibits Sestrin2-GATOR2 interaction. Arginine also promotes the activation of mTORC1 through binding to lysosome AA transporter SLC38A9 and CASTOR1. Activation of PI3K/Akt/Rheb signaling promotes the shuttling of PAT1 from the cell membrane to LEL membranes. PAT1 directionally transports AAs and protons out of the LELs coupled by the pumping of the protons back into the LEL lumen by v-ATPase, which subsequently leads to mTORC1 activation. Furthermore, AAs (perhaps glutamine and serine) also activate mTORC1 via stimulating interaction of AA transporter PAT4 with mTORC1 and its regulators Rab1A and Rheb on the Golgi. Arrows represent activation, whereas bars represent inhibition. AAs: amino acids; mTORC1: mammalian target of rapamycin complex 1; LELs: late endosomes and lysosomes; PAT1/4: proton-assisted AA transporters 1/4; LRS: leucyl-tRNA synthetase; CASTOR1: cellular arginine sensor for mTORC1; GATOR1/2: GAP activity towards Rags 1/2; PI3K: phosphatidylinositide 3-kinase; PKB/Akt: protein kinase B; Rheb: Ras homolog enriched in brain.

AAs should be transported into cells to be efficient activators of mTORC1 [26]. mTORC1 activation is completed by a lysosome-located small Ras GTPase Rheb [51,52]. There should be a close relationship between mTORC1 and lysosmes. The Rag GTPases, a sub-type of Ras-related small GTP-binding protein, have been reported as a regulator of TOR, and their activities could be affected by AAs [53]. There are four Rag proteins that work in heterodimers (Rag A/B combined with Rag C/D), and they interact with Raptor to transfer mTORC1 to lysosomes, which in turn leads to the activation of mTORC1 [8]. AAs determine the nucleotide loading of the Rag GTPases, which is crucial for the combination of Rag GTPases and Raptor. Rag GTPases interact with Ragulator and recruit it to lysosomes [11]. Ragulator is a pentameric complex consisting of proteins encoded by MAPKSP1, ROBLD3, C11orf59, C7orf59 and HBXIP [11,54]. Growing evidences suggest that AA sensing initiates at the lysosomes, and AAs must enter and accumulate in the lysosomes to initiate the signaling [55,56]. It seems that there must be one or more sensors at the lysosomes that can sense AAs and initiate mTORC1 signaling. The model of AA sensing by mTORC1 is indicated in Figure 3.

### 4.1. PAT1/SLC36A1

PAT1, another member of PATs, is a H^+^-coupled and pH gradient dependent AA transporter, which responds selectively to glycine, proline and alanine [57,58]. Goberdhan et al. [59] showed that PATs are potential regulators of cell growth. They found that mTORC1 activity and cell growth are decreased by the mutation of one PAT family member Pathetic (CG3424) and activated by the overexpression of either Pathetic or CG1139.

PAT1 was firstly identified in rat brain lysosomes. Thus, it is also called lysosomal AA transporter 1 (LYAAT1) [60]. In the study of a cell free system, it has been demonstrated that AA-induced mTORC1 activation initiates inside lysosomes [56]. Therefore, it is critical to understand the relationship between PAT1 and AA-induced mTORC1 activation. PAT1 knockdown in cancer cell lines and human embryonic kidney (HEK293) cells showed that PAT1 is critical for mTORC1-mediated cell growth [30]. However, according to the substrate specificity of PAT1, it can only transport some small, unbranched and neutral AAs, but not leucine and arginine, which can activate mTORC1 [57,58]. Moreover, Zoncu et al. [56] also suggested that overexpression of PAT1 completely suppresses the mTORC1 activation by AAs. All these findings indicate that it is not by the transport function that PAT1 influences mTORC1 signaling.

A recent report showed that PAT1 is primarily located on late endosomes and lysosomes (LELs) and acts as an essential mediator of AA-dependent mTORC1 activation by physically interacting with the Rag GTPases. Increased PI3K/Akt/Rheb signaling stimulates the endocytosis of PAT1 and synergistically enhances PAT-induced growth [61]. By combining these results and previous studies, Ögmundsdóttir et al. [61] put forward a model by which PAT1 participates in the regulation of mTORC1: PAT1 complexes with Rag/Ragulator/V-ATPase to form a “nutrisome” at the LEL membrane that may sense intralumenal AAs; PAT1 directionally transports AAs (perhaps glycine, proline and alanine) and protons out of the LELs coupled by the pumping of the protons back into the LEL lumen by v-ATPase, thereby leading to the activation of mTORC1 (Figure 3). Furthermore, the influx of leucine or other AAs into the LEL lumen may be required for the accumulation of the AA substrates for PAT1 in the LELs through AA exchange mechanisms, resulting in the PAT1-mediated activation of mTORC1. Of note, overexpressed PAT1 protein is unlikely to complex with Rag GTPases and merely accelerates the efflux of specific AA substrates for PAT1 out of LEL lumen [61], which may explain why high-level overexpression of PAT1 contrarily suppresses mTORC1.

### 4.2. SNAT9/SLC38A9

Zoncu et al. [56] revealed that AA sensing initiates at the lysosomes and requires the presence of AAs in the lysosomal lumen. Since then, researchers are concerned about whether any potential candidate proteins can act as AA sensors. Recently, human member 9 of the solute carrier family 38 (SLC38A9), a new lysosomal transmembrane protein, was found able to sense AAs and affect mTORC1 activity [55,62]. SLC38A9 was characterized as a low-affinity arginine transporter with a Ragulator-binding domain; depletion of SLC38A9 prevents mTORC1 activation in response to arginine but not leucine; and over-expression of SLC38A9 makes mTORC1 signaling insensitive to AA withdrawal but not to Rag activity [55]. Rebsamen et al. [62] showed that SLC38A9 is an effective transporter of glutamine and arginine, and demonstrated that SLC38A9 mediates glutamine efflux from proteoliposomes. Both studies indicate that SLC38A9 is a lysosomal transceptor and complexes with Rag-Ragulator to form an arginine sensor essential for mTORC1 activation.

### 4.3. LRS

Leucyl-tRNA synthetase (LRS) belongs to class I Aminoacyl-tRNA synthetases (ARSs), with a nucleotide-binding Rossmann fold, a large-insertion CP1 domain, an rRNA-binding anticodon domain, and a C-terminal extension domain [63]. ARSs catalyze the ligation of AAs to their cognate transfer RNAs (tRNAs) and can be classified into two classes [64]. Class I synthetases possess a nucleotide-binding Rossmann fold [65], whereas class II synthetases have different catalytic domains from Class I [63]. LRS has been shown as an AA sensor. Intracellular leucine combines with LRS and transfers it to lysosomes, which influences the nucleic acid binding state of Rag GTPases, and then regulates the localization and activation of mTORC1, while mutation of LRS desensitizes the mTORC1 pathway to AAs [66]. Bonfils et al. [67] studied the function of LRS in yeast and found that LRS senses leucine and combines with Gtr1 (the homologue of Rag A/B) but not with Gtr2 (the homologue of Rag D).

### 4.4. Sestrin2

Sestrin 1/2/3, a family of highly conserved stress-inducible proteins, play important roles in maintaining metabolic homeostasis and suppressing obesity- and age-associated pathologies [68,69]. Apart from redox regulation, Sestrins are also involved in stress-dependent mTORC1 regulation. It was previously reported that Sestrins specifically promote the activation of TSC2 by AMPK-mediated phosphorylation, and thereby inhibit mTORC1 [70,71]. More recently, Sestrin2 was also proposed to be a direct sensor of leucine for the mTORC1 pathway that activates RagA and RagB either directly or indirectly through GATOR2 (GAP activity towards Rags 2) [72,73]. Wolfson et al. [73] showed that the binding of leucine to Sestrin2 alters its melting temperature and inhibits its binding to GATOR2. Importantly, Sestrin2 physically binds to leucine with a binding affinity (*K*_d_) of 20 μM [73], which is lower than the Michaelis constant (*K*_m_) value of leucyl-tRNA synthetase for leucine (45 μM) [74]. Moreover, the structural basis for the sensing of leucine by the Sestrin2-mTORC1 pathway was further determined, and Sestrin2 was found to have a leucine-binding pocket which is necessary for the proper structural folding [72]. Structure-guided mutant of Sestrin2 with reduced affinity for leucine showed a dramatic increase of leucine concentration required for mTORC1 activation [72]. On the basis of these results, it can be speculated that Sestrin2 acts as a cytoplasmic leucine sensor, and leucine disrupts the Sestrin2-GATOR2 interaction by binding to Sestrin2, which in turn leads to lysosomal localization and activation of mTORC1 (Figure 3).

### 4.5. CASTOR1

The GATOR2-interacting cellular arginine sensor for mTORC1 (CASTOR1, previously named as GATS protein-like 3) has been recently reported as a cytosolic AA sensor for mTORC1 pathway [75,76]. The CASTOR1-mediated mechanism of AA sensing by mTORC1 is highly specific for arginine. CASTOR1 forms a homodimer and heterodimerizes with CASTOR2 to inhibit mTORC1 activity in the absence of arginine. Cytosolic arginine directly binds to CASTOR1 to disrupt the CASTOR1-GATOR2 interaction, resulting in the activation of mTORC1. Furthermore, the arginine-binding capacity of CASTOR1 is required for the activation of mTORC1 by arginine [75]. More recently, Saxton et al. [77] provided a structural basis for the sensing of arginine by mTORC1 pathway. They presented a 1.8 Å crystal structure of arginine-bound CASTOR1, and showed that the homodimeric CASTOR1 directly binds to arginine at the two ACT (Aspartate kinase, Chorismate mutase, TyrA) domains, leading to the dissociation of CASTOR1 from GATOR2 and activation of mTORC1.

## 5. Amino Acid Signaling to mTORC1 Initiates at the Golgi Apparatus

Classically, LELs are the platform for AA-dependent activation of mTORC1. However, two recent papers indicated that AAs can be sensed on the surface of the Golgi to regulate mTORC1 [50,78] (Figure 3). Rab1 has been previously known for its role in vesicular trafficking from the endoplasmic reticulum to the Golgi [79,80]. Thomas et al. [78] described that Rab1 functions as a conserved regulator that mediates AA signaling to mTORC1 via the Rag- and lysosome-independent mechanisms. Specifically, AAs promote Rab1A GTP binding and GTP-dependent interaction with mTORC1 and Rheb in the Golgi. Rab1A overexpression promotes mTORC1 signaling, causing stimulation of cell growth. Furthermore, PAT4, an AA transporter required for mTORC1 activation as described above, is also highly expressed on the trans-Golgi network in several cell types, suggesting the possibility that PAT4 might regulate mTORC1 activity in the Golgi [50]. Fan et al. [50] provided a model by which PAT4 interacts with both mTORC1 and its regulator Rab1A on the Golgi, which triggers the activation of mTORC1.

## 6. Amino Acid Signaling to mTORC1 Initiates at the Cell Membrane

As shown above, AAs should be firstly transported into the cell and then exert their signaling roles. However, recent researches revealed that without being transported into cells, AAs can also be sensed by membrane receptors and transmit the signal into the cell in a certain way. G protein-coupled receptors (GPCRs) are the largest membrane receptor family in mammal. GPCRs have ever been regarded as the receptors of hormones and neurotransmitters, while recent studies suggested that GPCRs can also sense nutrients. GPCRs are subdivided into three classes of A, B and C based on their sequence homology [81]. Of note, class C GPCRs are characterized by a large extracellular domain, which contains a nutrient sensing Venus Fly Trap domain (VFT) and a Cys-rich domain that couples nutrient binding in the VFT to the activation of G-proteins on the internal face of the 7-transmembrane domain motif [81]. This group includes classic metabotropic receptors for the neurotransmitter glutamate and a recently defined subgroup of broad-spectrum AA-sensing receptors including taste receptors (T1Rs), GPRC6A and the calcium sensing receptor (CaSR).

### 6.1. T1Rs

T1Rs consist of three sub-types: T1R1, T1R2 and T1R3, two of which combine to form specific heterogeneous dimmers T1R1/T1R3 and T1R2/T1R3. T1R1/T1R3 is an AA sensor and umami flavor detector [82,83], which can sense AAs and induce lysosomal localization and activation of mTORC1 [84], while T1R2/T1R3 is a sweet receptor and glucose sensor [85,86,87,88]. Owing to the species-specificity, T1R1/T1R3 in different animals can sense different AAs. Murine T1R1/T1R3 can sense up to 18 AAs [83]; human T1R1/T1R3 can sense only glutmate and aspartate [89]; while swine T1R1/T1R3 can sense at most 6 AAs, including glutmate, aspartate, alanine, glutamine, serine and threonine [90].

### 6.2. GPRC6A

Like the T1R1/T1R3 heterodimer, GPRC6A can be activated by basic, aliphatic and polar AAs, such as glycine, arginine, lysine, alanine and serine [91]. Besides, GPRC6A is also activated by extracellular calcium and other cations, calcimimetics and osteocalcin [92]. Coupled with Gi protein, GPRC6A could activate the ERK pathway in both cell culture and tissues [93]. Knockdown of GPRC6A inhibits the activation of ERK1/2, Akt and PKA, and inhibition of GPRC6A significantly decreases arginine-induced fibroblast proliferation in NIH 3T3 cells [94]. Importantly, activation of ERK1/2 and PI3K-Akt pathways promotes mTORC1 activation by directly phosphorylating Raptor [84,95] and enhancing Rheb-GTP activity [96], respectively. These findings leave open the possibility that GPRC6A may act as an AA sensor to regulate mTORC1.

## 7. Conclusions and Perspectives

As a central regulator of protein synthesis, mTORC1 has to integrate a wide range of intracellular and extracellular signals (including energy status, hormones, growth factors and nutrients such as AAs and glucose) to control protein translation, autophagy and cell growth. Being the basic substrate of protein synthesis, AAs can act as signal molecules and regulate the activities of a series of intracellular signaling pathways. Leucine, arginine and glutamine have been demonstrated to be effective activators of mTORC1, and we believe that more AAs with this role will be discovered. In the studies of the mechanism through which mTORC1 is regulated by AAs, more and more effectors were identified, including protein kinase, GTPases, AA transporters and AA receptors. Cutting off any one of them could decrease mTORC1 activity but not abolish the activation of mTORC1 completely, suggesting that mTORC1 signaling is not solely regulated by any of the above effectors. GTPases, AA transporters and AA receptors can sense different AAs, transmit the signal to mTORC1 by different signaling pathways, and maximize the activation of mTORC1. Furthermore, the current findings of mTORC1 activation at Golgi by Golgi-localized amino acid transporter PAT4 indicate a multi-hub model for mTORC1 control [97].

Despite this exciting progress, our understanding of AA-induced mTORC1 activation is far from complete, and there are still many questions to be resolved including the following: What is the mechanism by which glutamine is sensed and signals to mTORC1? What is the structural basis for the sensing of AAs by mTORC1? Can mTORC1 activity be stimulated at other organelles in addition to lysosomes and Golgi? Can the signaling pathways initiated by intracellular sensors and plasma membrane receptors of AAs together achieve the maximal activity of mTORC1? Further studies are required to address these questions, and thus to provide theoretical basis for developing novel therapeutic approaches to human diseases, particularly cancers, as well as for reasonably allocating nutrients.

## Abbreviations

mTOR	mammalian Target of Rapamycin
mTORC1	mTOR Complex 1
AAs	Amino Acids
Rag GTPases	Ras-Related Guanosine Triphosphatases
LAT1	System L AA Transporter 1
4F2hc	Heavy Chain of 4F2 Cell Surface Antigen
PATs	Proton-Assisted Transporters
LRS	Leucyl-tRNA Synthetase
SLC38A9	Human Member 9 of the Solute Carrier Family 38
GATOR1/2	GAP Activity towards Rags 1/2
CASTOR1	Cellular Arginine Sensor for mTORC1
GPCR	G protein-Coupled Receptor
S6K1	Ribosomal Protein S6 Kinase 1
4E-BP1	Eukaryotic Translation Initiation Factor 4E Binding Protein
mLST8/GβL	mammalian Lethal with Sec13 Protein 8
PRAS40	Proline-Rich Akt Substrate 40 kDa
Rheb	Ras-Homolog Enriched in Brain
TSC1/2	Tuberous Sclerosis Complex 1/2 Complex
PI3K	Phosphatidylinositide 3-Kinase
PKB/Akt	Protein Kinase B
RSK	p90 Ribosomal S6 Kinase 1
ERK1/2	Extracellular-Signal-Regulated Kinase 1/2
AMPK	AMP-Activated Protein Kinase
ASCT2	System ASC AA Transporter 2
SNAT2	Sodium-Coupled Neutral AA Transporter-2
LYAAT1	Lysosomal AA Transporter 1
LELs	Endosomes and Lysosomes
VFT	Venus Fly Trap Domain
T1Rs	Taste Receptor Family 1 Member
CaSR	Calcium Sensing Receptor
